# The glymphatic system in migraine and other headaches

**DOI:** 10.1186/s10194-024-01741-2

**Published:** 2024-03-11

**Authors:** Maria Grazia Vittorini, Aysenur Sahin, Antonin Trojan, Sevil Yusifli, Tamta Alashvili, Gonçalo V. Bonifácio, Ketevan Paposhvili, Viktoria Tischler, Christian Lampl, Simona Sacco, on behalf of the School of Advanced Studies of the European Headache Federation (EHF-SAS)

**Affiliations:** 1 Department of Biotechnological and Applied Clinical Sciences, University of L’Aquila, L’Aquila, Italy; 2 Faculty of Medicine-Acibadem Mehmet, Ali Aydinlar University, Istanbul, Turkey; 3 Department of Neurology, Strakonice Hospital, Strakonice, Czechia; 4 Faculty of Medicine-Istanbul University, Istanbul, Turkey; 5 Department of Internal Medicine, New Vision University Hospital, Tbilisi, Georgia; 6 Department of Neurology, Hospital de São Bernardo, Setúbal, Portugal; 7 Department of Neurology, Tbilisi State Medical University, Tbilisi, Georgia; 8 Department of Neurology, Konventhospital Barmherzige Brüder Linz, Linz, Austria

**Keywords:** Glymphatic system, Perivascular space, Cerebrospinal fluid, Neurological disorders, Migraine, Headache, Cortical spreading depression, CGRP, DTI-ALPS

## Abstract

Glymphatic system is an emerging pathway of removing metabolic waste products and toxic solutes from the brain tissue. It is made of a network of perivascular spaces, filled in cerebrospinal and interstitial fluid, encompassing penetrating and pial vessels and communicating with the subarachnoid space. It is separated from vessels by the blood brain barrier and from brain tissue by the endfeet of the astrocytes rich in aquaporin 4, a membrane protein which controls the water flow along the perivascular space. Animal models and magnetic resonance (MR) studies allowed to characterize the glymphatic system function and determine how its impairment could lead to numerous neurological disorders (e.g. Alzheimer’s disease, stroke, sleep disturbances, migraine, idiopathic normal pressure hydrocephalus). This review aims to summarize the role of the glymphatic system in the pathophysiology of migraine in order to provide new ways of approaching to this disease and to its therapy.

## Introduction

Migraine is a neurovascular disorder involving the trigeminovascular system [1]. It is one of the most frequent and disabling neurological diseases. It affects approximately 14% of the general population, mainly women, with a mean age of 15–40 years [2]. It represents a worldwide social and health concern [3]. Although the efforts in understanding migraine pathophysiology, the exact mechanisms underlying this disease still remain unclear. It has been hypothesized that the glymphatic system (GS) may play a role in migraine pathophysiology and that its disfunction may impact on the clinical spectrum of migraine. In this paper we reviewed current literature to summarize the available data concerning migraine, the GS and its involvement in migraine pathogenesis in order to provide a new insight into future diagnostic and therapeutic perspectives in the field of headache disorders.

## Migraine disease

Migraine is a complex neurovascular disorder involving the trigeminovascular system [1]. The current best estimate of global migraine prevalence is 14–15%, and, in terms of burden, migraine accounts for 4.9% of global ill health, quantified as years lived with disability [4]. Migraine causes negative consequences not only in patients immediately affected but also on their families, colleagues, employers, and society [5]. According to the International Classification of Headache disorders (ICHD) 3^rd^ edition, migraine could be divided into episodic migraine, with or without aura, and chronic migraine [6]. Migraine without aura is defined as a strictly unilateral, recurrent and pulsating pain, of moderate to severe intensity, lasting 4–72 h and accompanied by symptoms like nausea and/or vomiting, phono- and/or photophobia, aggravating by the body activity and alleviated by rest. Migraine with aura is characterized by the recurrence of reversible symptoms, preceding headache, which could be visual, sensory, speech/language, brainstem, motor or retinal disturbances. Which should show at least two of the following characteristics: spreading in 5 min and/or in succession, lasting 60 min, being unilateral and followed by headache associated with migraine symptoms. Chronic migraine is defined as a headache occurring on 15 days/month for more than 3 months, which has the features of migraine headache on at least 8 days per month [6].

Neuroinflammation, excess of calcitonin gene-related peptide (CGRP) and cortical spreading depression are the three most studied mechanisms underlying migraine pathogenesis and aura development. A common feature of these mechanisms is the impairment of the glymphatic system [2, 5, 7–23].

## The glymphatic system

**Fig. 1:**
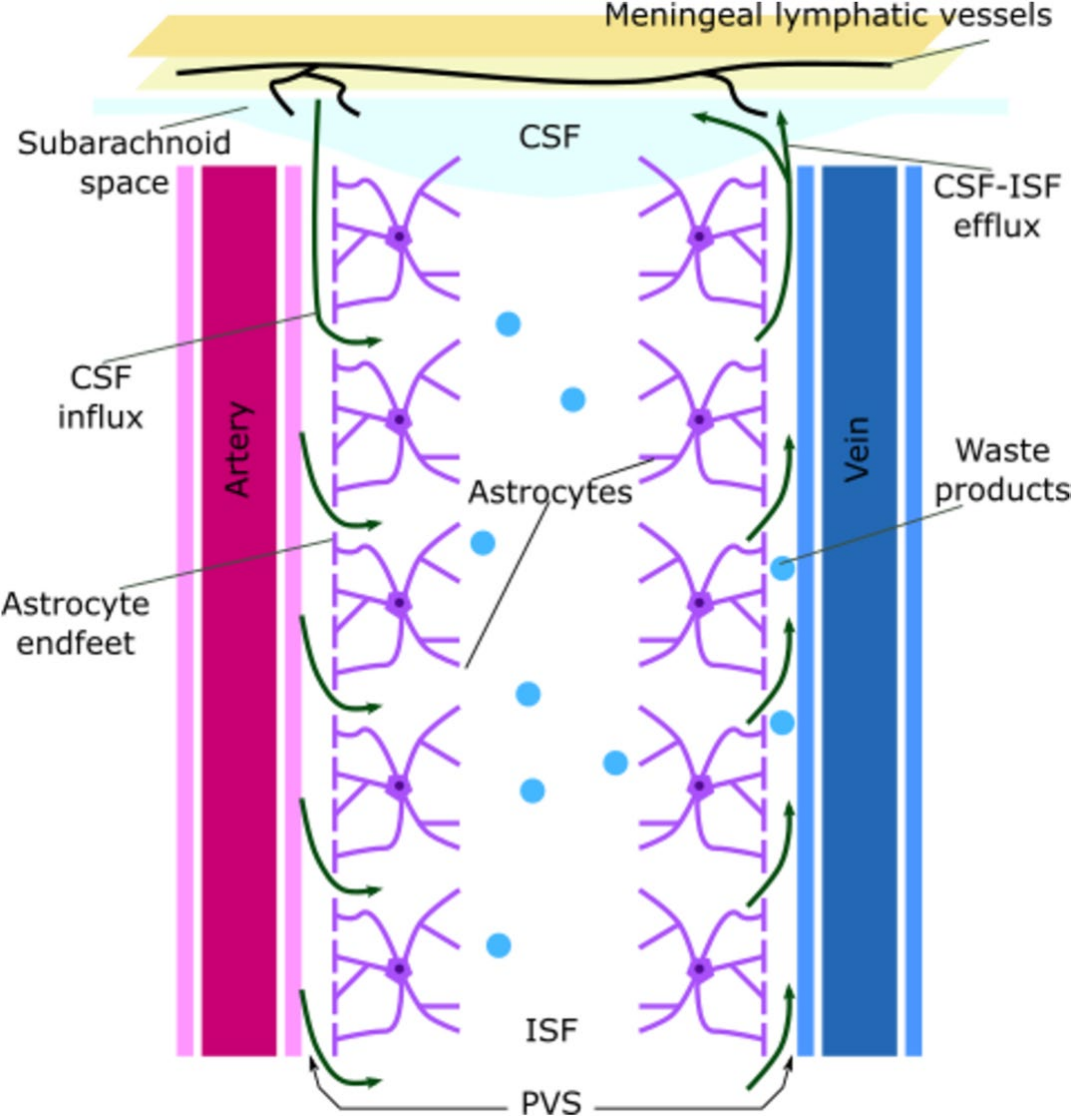
Representation of the glymphatic system and the glymphatic flow. The glymphatic system is made of a network of PVS around arteries and veins throwing metabolic waste products away from the central nervous system. PVS is limited by the endfeets of astrocytes and is filled with the CSF. CSF is produced in the choroid plexi in the lateral ventricles and then is vehicled into the subarachoid space. From the subarachoid space the CSF streams into the PVS around pial arteries. Here CSF enters the brain tissue and mixes with the ISF. CSF-ISF flows into the perivenous space and reach the dura mater sinuses, the meningeal lymphatics and the cervical lymphnodes. PVS: perivascular space; CSF: cerebrospinal fluid; ISF: interstitial fluid

The lymphatic system, a vast network of vessels and lymphoid organs, assures intrabody fluid homeostasis and immunity by collecting and detoxifying fluid and metabolic waste products in the interstitial space [24, 25]. Although the brain tissue is among the most metabolically active organs of the body, there is no classical lymphatic circulation clearing its metabolites and waste products [26]. Nonetheless, recent studies have demonstrated the existence of the so called glymphatic system (GS). It is a complex network of perivascular space (PVS) surrounding brain vessels and acting as a possible lymphatic circulation. The outer perimeter of the PVS is lined with foot-like protrusions of astrocyte cells [27] and is separated from the vascular wall by a basement membrane called glia limitans [28]. PVS is filled with the cerebrospinal fluid (CSF) [1]. CSF is produced by the choroid plexi located in the brain ventricles, through ${{\text{Na}}}^{+}$/${K}^{+}$ ATP-ase and Aquaporin 1 [29]. Despite CSF shares many similarities with blood plasma, it has higher levels of sodium, chloride, and magnesium, and lower levels of potassium, calcium, proteins, and cells [30]. After its synthesis CSF spreads into the ventricles, the subarachnoid space and the PVSs. It enters the brain tissue through para-arterial space and mixes with the interstitial fluid (ISF). CSF-ISF complex and its solutes enter the paravenous space thanks to the water transporter Aquaporin 4 (AQP4) sited into the astrocytes’ endfeet [31]. Once CSF-ISF has reached the subarachnoid space, it passes through the arachnoid granulations into the dural sinuses, the meningeal lymphatics and into the cervical lymphatics [32, 33] (Fig. 1).

The GS is involved in the drainage of metabolic waste products such as lactic acid, tau protein, Amyloid-β or α-Synuclein. It plays a pivotal role in the exchange of nutrients (like glucose and lipids), neurotransmitters, and neuroactive substances (such as transretin and apoprotein E) [34, 35]. The flow within the GS could be influenced by factors such as changes in the arteriovenous hydrostatic gradient, vascular vasodilation or vasoconstriction, body position, circadian rhythm, respiration, heart rate and intracranial pressure [26, 36, 37].

## Glymphatic dysfunction in migraine

**Fig. 2:**
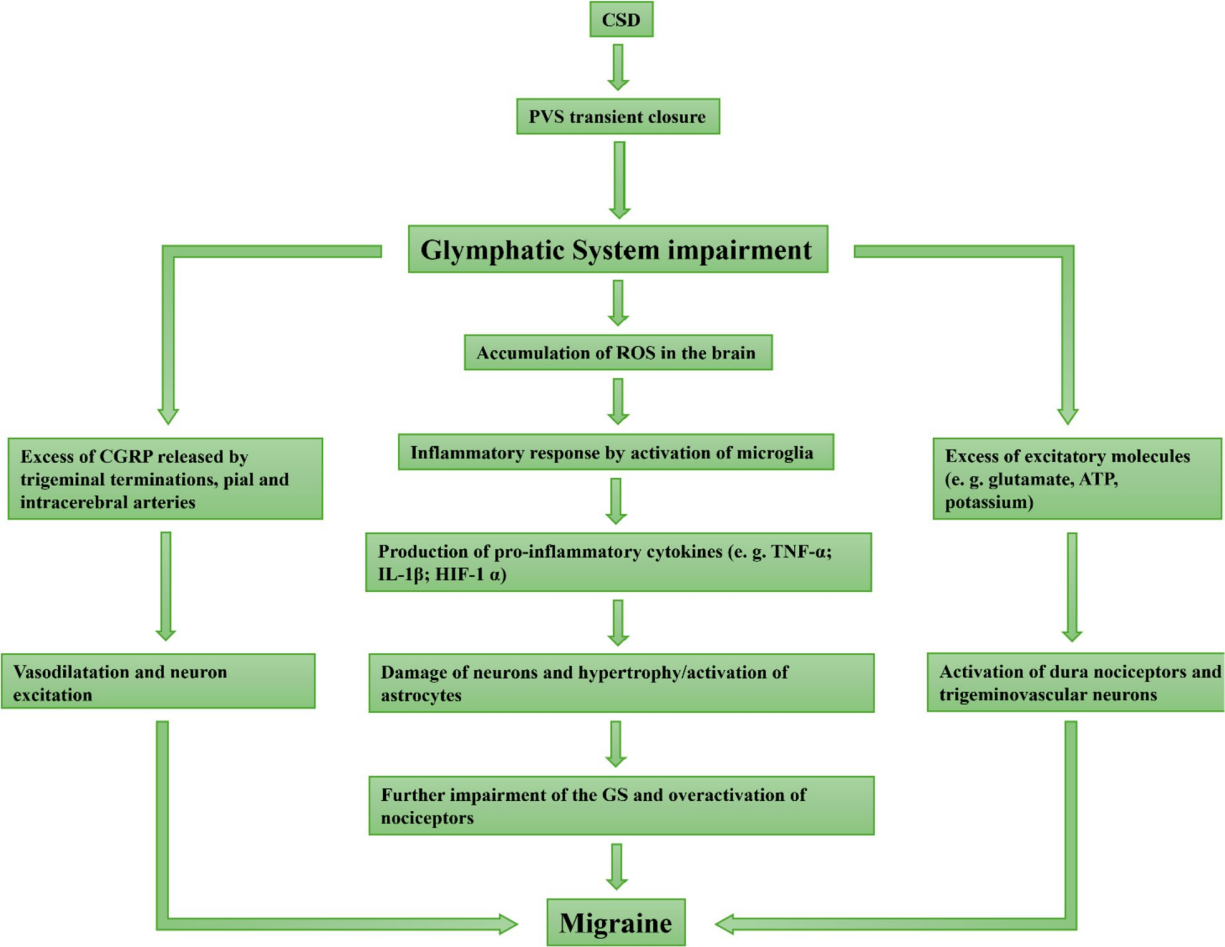
Possible pathogenetic mechanisms of migraine involving the glymphatic system dysfunction. CSD can be responsible for a transient PVS closure causing a GS impairment. The GS dysfunction can lead to the accumulation of excitatory, pro-inflammatory and vasodilator molecules involved in the development and the exacerbation of migraine. CSD: cortical spreading depression; PVS: perivascular space; ROS: reactive oxygen species; CGRP: calcitonin gene-related peptide; ATP: adenosine triphosphate; TNF-α: tumor necrosis factor alpha; IL-1β: interleukin 1-beta; HIF-1α: hypoxia-inducible factor 1-alpha

It has been postulated that the GS could contribute to the pathogenesis of migraine. Even though the exact mechanism underlying this relationship remains to be fully elucidated, three main potential mechanisms have been hypotesized: neuroinflammation, calcitonin gene-related peptide (CGRP) dysregulation and cortical spreading depression (CSD) (Fig. 2).

Regarding neuroinflammation, it is known that GS is crucial for removing reactive oxygen species (ROS) [5]. Brain tissue is extremely susceptible to ROS damage due to its high oxygen consumption, lipidic metabolism and poor antioxidants [7]. An excess of ROS in the brain activates the immune response by the microglia production of proinflammatory cytokines such as tumor necrosis factor alfa (TNF α), interleukin 1beta (IL-1β) and HIF-1α [8, 9]. These molecules are released into the extracellular space (ECS) and flow in the glymphatic network. Thus, an impairment of the GS results in the accumulation of proinflammatory cytokines and ROS leading to the degeneration of neurons and to the hypertrophy and activation of astrocytes [10, 11]. The disruption of the astrocytes further aggravates the GS dysfunction and starts a vicious circle of events [12]. Neuroinflammation has been enquired as a possible mechanism linking the GS dysfunction with migraine development. It is known that pro-inflammatory cytokines can exacerbate nociceptive stimuli overactivating neurons and nociceptors [13–16].

CGRP is a key player in the pathogenesis of migraine. It is a neuropeptide produced both in the central and in the peripheral nervous system [17]. CGRP exerts a vasodilator action on blood vessels and acts as a neuron excitability modulator [18]. After its release from trigeminal terminations of meninges, pia mater and intracerebral arteries, CGRP doesn’t cross the blood–brain barrier but could rapidly penetrate the PVS encompassing pial artery [19]. Within the CSF-ISF, it reaches the perivenous space and hence the bigger dura mater sinus. Then, the final step are the lymph nodes of the general lymphatic system [2]. It can been postulated that an impairment of the GS may increase CGRP concentration and thus worsen migraine.

Finally, studies investigated the role of CSD as an additional hypothetic mechanism explaining how the GS dysfunction could lead to the development of migraine [2, 19–23]. CSD is a chemo-electrical excitatory wave propagating across the brain surface. It is supposed to be the major pathophysiological mechanism of migraine aura. When a CSD-wavefront arises, the physiological ions concentration in the CSF changes: ${\text{Na}}^{+}$ and ${\text{Ca}}^{2+}$ enter into the cells, while ${\text{K}}^{+}$ flows out of neurons. In physiological conditions, neurons maintain a potential of -70 mV thanks to ions channels and membrane pumps. Depolarization of cells membrane occurs when the relative excess of ${\text{K}}^{+}$, adenosine triphosphate and hydrogen ions in the ECS makes their potential achieve the value of approximately -10 mV [19, 21]. CSD can cause a succession of vasoconstriction and vasodilatation of the pial and penetrating arteries, thus modifying the PVS radium and interfering with the normal function of the GS. Moreover, this change in ionic distribution leads to a swelling of neurons whose activity remains temporarily suppressed [20, 21, 23]. At the end of the CSD the excess of ${\text{K}}^{+}$ is removed both through the CSF and via ${\text{K}}^{+}$ buffers [20]. Since those evidence is based on studies in the experimental animal, further research is needed to extend these findings to the human [2, 20, 22].

## Techniques for investigating the glymphatic system

In animal models, optical imaging techniques, particularly two-photon microscopy, have traditionally held a prominent role in the study of the GS. This is primarily due to their exceptional spatial resolution, which is crucial for capturing tiny PVS. In in vivo studies, after the intracisternal injection of small fluorescent tracers, in anesthetized mice, two-photon microscopy has been used to determine the dynamics and the anatomic structure of the glymphatic flow [31, 37–40]. However, two-photon microscopy doesn’t allow to visualize the deeper regions of brain and for this reason ex vivo studies have been employed to analyze the GS distribution and function in the whole brain or in specific regions [31, 39]. In ex vivo experiments, coronal or sagittal slices of death mice brains injected with fluorescent tracers are visualized under a microscope [41, 42]. To quantitatively determine the fluorescence distribution in the slices an imaging processing is needed: a whole brain slice or a region of interest is chosen and the mean pixel intensity or the coverage area of the fluorescent tracer is manually analyzed in imaging software [31, 41, 43, 44] However, this technique is time consuming and, more important, the fluorescence of the slices doesn’t always faithfully depict the distribution of tracers in the live brains. For these reasons, the use of spectrophotofluorometry on microplate assays is preferred to better quantify the distribution of fluorescent tracers in animal brain slices as a marker of the GS function [39].

MR imaging has been used in animal models to visualize the distribution and to characterize the function of the GS [45, 46]. Iliff et al*.* used a gadolinium-based contrast agent to describe the GS flow into the brains of living rats [47].

While direct measurement techniques utilizing fluorescence and contrast agent tracers can be used in animal studies, their application in humans is invasive and comes with inherent challenges. Consequently, there is a pressing need to explore alternative noninvasive methods that facilitate the study of the glymphatic system in human subjects [48, 49]. So far, no ideal technique is available to study the GS in humans but several techniques have been employed as tools to provide different type of information on the GS function in humans. Research studies employed MR, positron emission tomography and ultrasound [50–53].

MR offers distinct advantages, including the ability to overcome the limited penetration depth of two-photon microscopy and the capacity to perform whole-brain imaging, in contrast to two-photon microscopy [38, 50]. Several noninvasive MR methodologies offer the opportunity to investigate the dynamics of ISF and CSF flow within the cerebral tissue in human subject: T1 and T2 weighted sequences, T2 fluid-attenuated inversion recovery (FLAIR); PVS imaging; dynamic contrast-enhanced MR imaging (DCE-MRI); diffusion tensor image analysis along the PVS (DTI- ALPS), arterial spin labeling, chemical exchange saturation transfer, and intravoxel incoherent motion [49].

PVS exhibits hyperintensity on T2-weighted imaging, isointensity on proton density weighted imaging and hypointensity on T1 and T2 weighted imaging and FLAIR. The abnormalities of PVS can be detected as ectatic and less regular spaces at MRI. The combination of T1 and T2 weighted imaging, as well as T1 and FLAIR can enhance the sensitivity of PVS identification [34, 54–56].

A common tool to analyze the distribution of PVS in human brains is a visual rating scale based on the Potter scoring which grades PVSs from 0 to 4 according to their numbers in the brain plane calculated in the basal ganglia and in the centrum semiovale on structural brain imaging. Potter scoring also grades midbrain PVSs 0–1 according to their presence or absence [49, 57, 58]. However, this technique is influenced by the experience of observers and by the ceiling effect which affect the inter- and intrareproducibility [49]. For these reasons, Dubost et al. developed an automatic system of grading PVS at MRI in humans [59].

DCE-MRI measures the movement of contrast agents in the ISF within the PVS and brain tissue, eliminating the necessity for intricate modeling or postprocessing techniques. Additionally, intrathecal DCE-MRI has the potential to achieve precise and detailed spatial resolution. The extensive use of DCE-MRI for investigating ISF properties in humans is hampered by several factors. The procedure is invasive, requiring sterile conditions and the expertise of healthcare professionals, it can be uncomfortable for patients and can be biased by limited spatial and temporal resolution of tracers and by movement artifacts [38, 60]. Furthermore, intrathecal injections of gadolinium-based contrast agents may lead to adverse reactions, such as anaphylactic responses, including headache and severe nausea [61], and carry the risk of nephrotoxicity, potentially causing renal failure [62], as well as neurotoxic effects, which may manifest as speech issues, psychotic symptoms, lethargy, and visual impairment [63]. There is also the concern that gadolinium can enter brain tissue through the glymphatic system and deposit in parenchymal tissues, including the dentate nucleus and globus pallidus [64, 65].

DTI-ALPS is a method that utilizes diffusion MR to assess the activity of the glymphatic system by examining the dynamics of ISF within the human brain. This technique entails the analysis of DTI along the PVS, and the outcomes are represented as ALPS scores. When the ALPS index approaches a value of one, it indicates that water diffusion within the PVS has a minimal impact. In contrast, a higher ratio suggests an elevated level of water diffusivity within the PVS. The method proposed for computing the ALPS index using DTI-ALPS is influenced by head rotation, potentially leading to reduced reproducibility and reliability. To address this issue, an additional approach involving DTI reorientation was introduced for ALPS index calculation based on DTI-ALPS. Taoka et al. also proposed a method involving the utilization of diffusion-weighted imaging with a three-axis diffusion gradient direction for the computation of the ALPS index within the framework of diffusion-weighted imaging-ALPS [66].

Arterial spin labeling, chemical exchange saturation transfer and intravoxel incoherent motion are new promising MRI technique that indirect assess the GS function by the analysis of blood–brain barrier permeability, by the estimation of solutes at two order of magnitude lower concentration than traditional MRI and by the evaluation of the diffusion/perfusion effect of blood motion in tiny vessels [49].

Ultra-high field MR imaging, acquired at ≥ 7 T, is a valuable approach to better detect PVS abnormalities in human subjects [67–69]. However, there are some limitations of this technique: ultra-high MRI is more sensitive to movement artifacts, there is a magnetic field dishomogeneity causing a higher difficulty to identify subcortical PVS, there are issues about the radiofrequency absorption rate and a more limited compatibility of medical devices with magnetic coils [34].

Positron emission tomography and ultrasound imaging are two further noninvasive imaging techniques which could be used to investigate the GS [38, 52, 53].

Further details are shown in Table 1.

**Table 1:** Investigation of the glymphatic system: different imaging techniques with their advantages and disadvantages

Investigation tool		Technique	Pros	Cons
**Imaging**	Optical imaging	Two-photon microscopy	Tracking tracers with high spatial and temporal resolution	Limited field of view Invasive technique
	MRI	FLAIR	Facilitates the visualization of perivascular spaces and the assessment of glymphatic function	Low specificity
		CE-MRI and DCE-MRI	Noninvasive technique Whole brain images 3D visualization	Low spatial and temporal resolution Movement artifacts
		DTI-ALPS	Quantify the diffusion of water molecules within the brain's interstitial space, providing valuable insights into glymphatic function	Low specificity
		Arterial spin labeling, chemical exchange saturation transfer, intravoxel incoherent motion	Blood brain barrier permeability, assess of solutes concentration at two order of magnitude lower than traditional MRI, diffusion/perfusion effect evaluation of blood motion	Indirect measurements of the glymphatic system function Need of integration with other techniques
		ULTRA-HIGH MRI	Better detection of PVS abnormalities	Movement artifacts, dishomogeneity in magnetic field, difficulty to identify subcortical PVS, radiofrequency absorption rate and lower compatibility of medical devices
	Positron emission tomography	Use of radiolabeled tracers	Whole brain images Quantify the glymphatic system clearence	Low spatial resolution Movement artifacts
	Ultrasound Imaging	Transcranial Doppler ultrasound	Study glymphatic pulsations and CSF dynamics in humans	Low specificity

*Abbreviations:* MRI Magnetic Resonance Imaging, *FLAIR* Fluid-Attenuated Inversion Recovery, *CE/DCE MRI* Contrast Enhanced/Dynamic Contrast Enhanced Magnetic Resonance Imaging, *DTI* Diffusion Tensor Imaging, *PVS* perivascular space, *CSF* Cerebrospinal fluid

## Animal models

There are some studies aimed to elucidate the way in which the GS could be involved in the pathophysiology of migraine in the animal model of this disease [37, 70].

Huang et al. used a nitroglycerin (NTG)-induced migraine mouse model to clarify whether the GS dysfunction is a trigger or an aggravating factor of migraine [70]. The NTG administration in adult mice determined a decrease in the mechanical pain threshold and the stimulation of meningeal nociceptors by the release of nitric oxide, when compared with healthy controls. Using immunofluorescence, Huang et al. demonstrated that there is a reduction in the AQP4 expression in the PVS in mice with NTG induced migraine, especially in those simultaneously treated with an AQP4 inhibitor (TGN-020). The changes in AQP4 distribution led to a massive release of CGRP [70]. Immunofluorescence in NTG-induced migraine models further revealed that TGN-020 administration determined an increased expression of c-Fos as a marker of increased neuronal activity as well as an increase in astrocytes and microglia activation as a marker of neuroinflammation in the medullary dorsal horn. NTG-induced migraine models simultaneously treated with TGN-020 showed a reduction in the distribution of a tracer (TR-d3) when it is injected in the cisterna magna. Taken together these results suggest that the GS dysfunction is an aggravator factor rather than a trigger mechanism of migraine [70].

Schain et al. used an in vivo two-photon imaging technique to determine whether CSD alters the function of the GS in mice models [37]. Using dying tracers they demonstrated that PVS system is a wide, cleansing network encompassing both superficial and penetrating vessels (arteries and veins) and that it is bordered by endothelium, pia mater and brain tissue. PVS diameter is influenced by anatomy: it is larger in case of multiple vessels and of vessels bifurcations. Orthogonal reconstruction of superficial vessels was used to quantify the tridimensional rate between PVS width, vessel lumen and subarachnoid space. This technique was less efficient when used to determine PVS diameter in penetrating vessels. Inducing CSD by pinprick in the brain cortex of non-injected mice, Schain et al. observed that CSD causes an initial constriction of superficial vessels (arteries and veins), followed by a dilatation at 3 min from the beginning of the stimulation and by a final constriction that lasts for about 22 min. CSD causes the complete closure of PVS at 6 min after its induction. Thereafter, PVS slowly reopens but remains partially closed for about 30 min. It still remains unknown the exact mechanism underlying the closure of PVS during CSD but some studies hypothesize that it depends on the swelling of neurons and astrocytic endfeet during CSD [71, 72]. After the injection of tracers, Schain et al. demonstrated that they accumulate into the PVS after the arrival of CSD wavefront and that the glymphatic flow is delayed and slowed [37]. This confirms the hypothesis that CSD causes the storage of excitatory and neuroinflammatory chemicals such as glutamate [73], ATP [74] and potassium [75, 76] in the PVS. At the end of CSD, when PVS reopens, all the excitatory molecules reach and activate the dura nociceptors and central trigeminovascular neurons [77, 78]. This sequence of events sems also to explain the delayed onset of pain in patients suffering from migraine with aura [37].

In summary, experimental studies investigating the glymphatic system in migraine are so far limited. Available data indicate that the GS dysfunction acts more consistently as an aggravator rather than as a causal factor of migraine. Furthermore, it has been demonstrated that a transient closure of the PVS is involved in the development of CSD, the most investigated pathogenetic mechanism of migraine aura, by the accumulation of pro-inflammatory and irritative molecules. These molecules than contribute to the activation of the trigeminovascular nociceptors determining headache pain.

## Human findings

Few studies have investigated the role of the GS dysfunction in migraine in humans [2, 79–94].

A recent pilot study using the DTI-ALPS index compared healthy controls with people with migraine, both with and without aura. It demonstrated that there is not a significant difference in the DTI-ALPS index, as a marker of the GS dysfunction, in the two examined groups and also between individuals with and without aura. These findings suggest a weak engagement of the GS impairment in the pathophysiology of migraine, but further research is needed to confirm these observations [80].

Another possible marker of the GS dysfunction are the enlarged perivascular spaces at MRI. Using a 3 T MRI technique, Yuan et al*.* investigated the correlation between enlarged perivascular spaces, as a marker of GS dysfunction, and migraine in three groups: healthy controls, episodic migraine and chronic migraine. They showed that an increase in the PVS width, especially in the centrum semiovale and in the midbrain, is an independent predictor factor of migraine. In this same study GS dysfunction was not associated with the clinical manifestation and the chronification of migraine [2].

To evaluate whether the GS activity changes during chronification of migraine, Zhang et al. used the DTI-ALPS index on a cohort of people with CM. They compared the results obtained in the CM group with those emerged from healthy controls and from episodic migraine group. During migraine chronification, the DTI-ALPS score is improved rather than diminished [81]. The raise of the DTI-ALPS index in CM seems to be related to the alteration of vascular reactivity induced by the prolonged release of CGRP during each migraine attack. CGRP, in fact, causes a central sensitization thought to be the mechanism underlying migraine chronification. Furthermore, Zhang et al. demonstrated that the improvement of the DTI-ALPS index lateralized on the right hemisphere in CM [81], confirming the results of previous studies investigating the lateralized manifestation of headaches [82–86]. Functional MR imaging studies hypothesized that the predominance of the right hemisphere dysfunction in headaches disorders could depend on abnormal connections between the right thalamus and some ipsilateral cortical regions involved in the regulation of pain (primary somatosensory cortex and premotor cortex) [85]. MR spectroscopy further confirmed that the right thalamus of migraineurs has increased levels of glutamate and glutamine [86]. These findings suggest that in CM there is an improvement of the GS activity. Zhang et al*.* hypothesized that the GS overactivation during migraine chronification could be a concomitant phenomenon of the vascular reactivity induced by an accumulation of CGRP in the interictal period starting the mechanism of central sensitization. However, this study has some limitations: first, the number of participants was too small to extend the results to the whole population of individuals with CM; second, the DTI-ALPS score, as a marker of the GS activity, is commonly calculated on slices of the lateral ventricle body and so it represents partial function of the entire GS; third, data on the CGRP levels in the brain of the participants were not collected. For these reasons, further researches are needed to validate these findings [81]. More recently, Wu et al. study used MRI techniques to establish whether the GS and the meningeal lymphatic vessels function are altered in people with chronic migraine (both with and without analgesic medical overuse) and episodic migraine, compared with healthy controls. They demonstrated a negative correlation between the DTI-ALPS index in chronic migraine, especially in people with a medical overuse, rather the in episodic migraine or in healthy control, which suggest a dysfunction of the GS in chronic migraine. Furthermore, they observed a negative correlation also between the DTI-ALPS and migraine disability, especially when migraine attacks frequence was > 4 per month. Additionally, they observed that a negative correlation exists also between the DCE-MRI values of time to peak, mean time to enhance, enhancement integral and chronic migraine, suggesting an impairment of the meningeal lymphatic system in chronic migraine [87].

Some studies have provided evidence that cerebral small vessel disease may be associated with a dysfunction of the GS [88]. Following these findings, Ornello et al. hypothesized that GS dysfunction may contribute to the development of white matter hyperintensities (WMHs) in people with migraine [89, 90]. WMHs are a common finding at MR in individuals with migraine but their nature is still unclear [88, 91]. It is supposed that WMHs represent an expression of subtle ischemic suffering of brain tissue caused by an impairment of normal perivascular outflow of CSF-ISF which, in turn, leads to an accumulation of waste products into the ECS [92]. It has been suggested that CSD, the surrogate of migraine aura could determine, through the vasoconstriction of pial and penetrating arteries, a spreading ischemia and the appearance of WMHs at MR [93]. For these reasons, it could be postulated that the WMHs in patients suffering from migraine represent a consequence of the dysfunction of the GS [94]. To evaluate whether WMHs in migraineurs are associated with the GS dysfunction, Ornello et al. used the DTI-ALPS index. Using this technique, the authors did not find that GS dysfunction was associated with WMHs in patients with migraine [94].

To summarize, human studies came with conflictual data regarding the involvement of the GS dysfunction in migraine pathogenesis, especially in people suffering from chronic migraine. Some limitations occurred in the studies design making results not generalizable to the whole population of individuals with migraine. For these reasons, further research is needed to fully elucidate the role of the GS in migraine development as well as to define new techniques of investigating the GS changes in humans suffering from migraine. To move the field forward it would be important to investigate well defined populations, harmonizing methods and possibly using multicenter study design.

## Other headache disorders and the glymphatic system

**Fig. 3:**
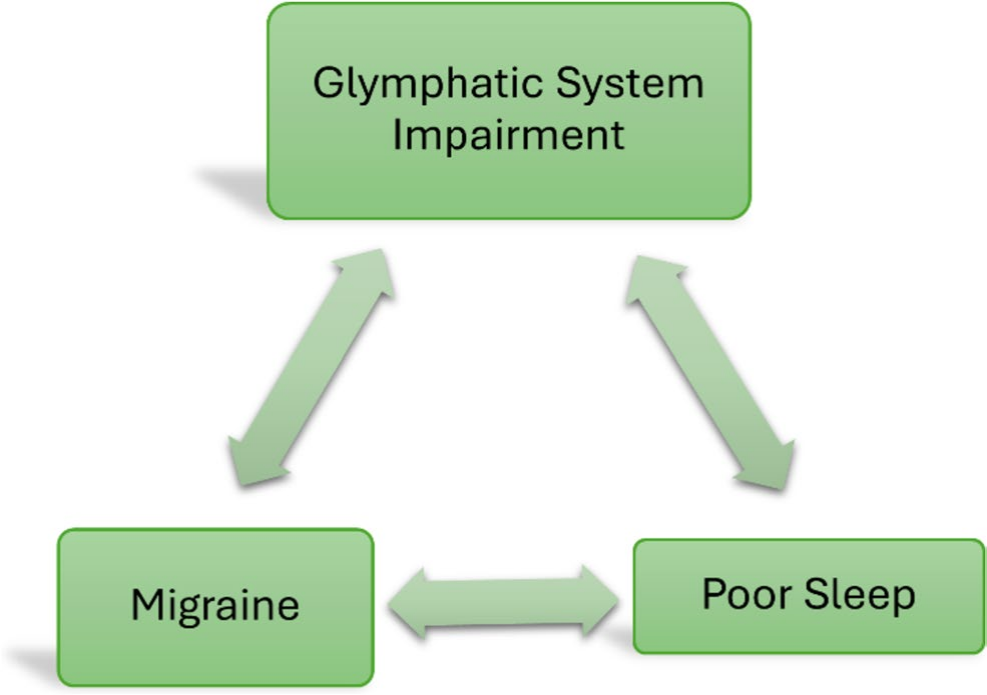
Relationship between glymphatic system impairment, sleep disturbance and headache. Poor sleep can determine an impairment in the glymphatic flow which, in turn, leads to the accumulation of neuroexcitatory and pro-inflammatory chemicals involved in the development of headache. Headache itself can, directly or indirectly (via a dysfunction of the glymphatic system) exacerbate sleep disturbances

Several studies have reported that there is an association between sleep disturbances and impairment of the GS. Since sleep disturbances are common in migraine and may be present also in other headache disorders it can be postulated that there is an interconnection among all those conditions. In recent years, it has been pointed out the possible bidirectional involvement of sleep abnormalities and GS [95–99]. Animal models demonstrate that the flow into the GS is facilitated during the sleep, especially during the deep sleep [96, 97]. Thus, sleep disturbances can diminish the efficacy of the GS in recycling metabolic waste products, resulting in a greater likelihood of developing migraine and dementia [95, 98] (Fig. 3). Furthermore, awakening causes a greater production of norephinepfrine which reduces the interstitial space and determines an accumulation of molecules involved in the pathogenesis of vary neurological diseases [99]. GS disfunction can also lead to the accumulation of orexin (both A and B) in the brain tissue, especially in the dorsal raphe and in the locus coeruleus causing sleep fragmentation and inefficiency [95].

Cluster headache (CH) is considered the most painful type of headache [35] and according to the ICHD-3rd edition it is defined as a strictly unilateral orbital, supraorbital or temporal pain that lasts for about 15–80 min and occurs for 1–8 times per day. It is associated with autonomic ipsilateral manifestations such as tearing, conjunctival injection, eyelid edema, excessive sweating, rhinorrhea, miosis, ptosis and agitation [6]. Kim et al. investigated the possible association between GS impairment and CH, focusing on the role of sleep disturbances and ageing. Using the DTI-ALPS index they demonstrated that patients with CH show a decreased activity of the GS and they identified a negative correlation between the DTI-ALPS index reduction in CH and age. These findings confirm the existence of a bidirectional link between CH and sleep disturbances in aged brains: ageing reduces the total time spent in deep sleep causing a decreased removal of waste products from the brain, included those involved in the development of CH; on the other hand, CH causes sleep fragmentation and the accumulation of molecules involved in different neurological diseases (e.g. β-amyloid, tau protein, pro-inflammatory cytokines) [24, 25, 100–103].

The GS inefficiency has been correlated also with the development of idiopathic intracranial hypertension (IIH) [104]. It represents an augmentation of the normal intracranial pressure (ICP) that, in normal conditions, is determined by the balance of three compounds: CSF, brain tissue and blood vasculature. Considering that the skull is a fixed volume, the relative excess of one of these three compounds is sufficient to cause an increment in the intracranial pressure, determining symptoms like nausea, vomiting, tinnitus, headache, and visual impairment. When the glymphatic flow is diminished, an accumulation of CSF into brain tissue and nerves sheaths (particularly in the optic nerve) happens and leads to the typical radiological signs of IIH: congestion of the GS and overflow of CSF into the lymphatic pathway [104–106]. The third radiological sign of IIH, the venous stenosis, both intrinsic and extrinsic [107], is typical in symptomatic patients with papilledema and increased ICP. Particularly, transverse sinus stenosis determines an impairment of the CSF outflow via the venous system. Thus, CSF drainage, as well as ICP, become largely dependent on the efficiency of the glymphatic pathway. For these reasons, ICP in IIH could be extremely variable and this variability could explain why some individuals with chronic migraine, also vestibular migraine, and isolated tinnitus could show radiological signs of IIH without meeting the diagnostic criteria for IIH (Dandy’s criteria), based on the presence of high ICP [108, 109]. Vice versa, increased ICP and papilledema could represent a severe stage of IIH [104]. These observations suggest the need to upgrade Dandy’s criteria for the diagnosis of IIH, introducing the radiological signs as diagnostic criteria and reducing the importance of increased ICP [104].

Traumatic brain injury is frequently associated with the development of post-traumatic headache and chronic migraine via an impairment of the GS [95]. Studies reported that traumatic brain injury causes a regional brain damage that leads to a disruption of the GS with the consequently accumulation of solutes and neurotoxic molecules, as far as excitatory products that feedforward the headache [110].

## Conclusions

The study of the glymphatic system is giving more insights on the mechanisms underlying several neurological disorders and particularly sleep, neuroinflammatory and neurodegenerative diseases. Despite a rationale and experimental studies supporting a possible involvement of the glymphatic system in migraine and in other headache disorders so far no conclusions in this regard can be reached. The human studies investigating the glymphatic system in migraine and in other headache disorders are limited and mostly lead to negative results. To move the filed forwards, further improvements in techniques to investigate the glymphatic system in vivo in humans are needed as well as proper designed studies that consider possible confounders and contributing factors.

## Data Availability

No datasets were generated or analysed during the current study.

## Abbreviations

GS: Glymphatic system
PVS: Perivascular space
CSF: Cerebrospinal fluid
ISF: Interstitial fluid
AQP4: Aquaporin 4
MRI: Magnetic resonance imaging
FLAIR: Fluid attenuated inversion recovery
DCE-MRI: Dynamic contrast enhancement magnetic resonance imaging
DTI-ALPS: Diffusion Tensor Imaging Along the Perivascular Space
ROS: Reactive oxygen species
TNF-α: Tumor Necrosis Factor alpha
IL-1β: Interleukin 1-beta
HIF-1α: Hypoxia-inducible factor 1-alpha
CGRP: Calcitonin Gene-related Peptide
CSD: Cortical spreading depression
NTG: Nitroglycerin
ATP: Adenosine triphosphate
WMH: White matter hyperintensity
CH: Cluster headache
IIH: Idiopatic intracranial hypertension
ICP: Intracranial pressure
